# Evaluation of *EGR1* as a candidate gene for high myopia

**Published:** 2008-07-15

**Authors:** Tuo Li, Xueshan Xiao, Shiqiang Li, Yiqiao Xing, Xiangming Guo, Qingjiong Zhang

**Affiliations:** 1 Department of Ophthalmology, Renmin Hospital, Wuhan University, Wuhan, China; 2 State Key Laboratory of Ophthalmology, Zhongshan Ophthalmic Center, Sun Yat-sen University, Guangzhou, China

## Abstract

**Purpose:**

*EGR1* (OMIM 128990) is an early growth response gene that has been shown to be related to myopia by upregulation and down-regulation of axial eye growth in experimentally induced chicken myopia and knockout mice. The purpose of this study was to test whether variations in the human *EGR1* gene are related to high myopia.

**Methods:**

Genomic DNA was prepared from leukocytes of peripheral blood. Cycle sequencing was used to detect sequence variations in *EGR1*.

**Results:**

No pathological mutations were detected upon sequencing of the coding regions and the adjacent intronic regions of *EGR1* in 96 unrelated Chinese subjects with high myopia. Only one silent variation, c.1026G>A (p.V342V), was detected in one patients with high myopia, which would not affect the encoded protein. For all 96 subjects, only one allele was detected in each of the three known single nucleotide polymorphisms (SNPs) in *EGR1*.

**Conclusions:**

We found no evidence that *EGR1* is responsible for high myopia in these patients.

## Introduction

Myopia is a leading visual problem that affects an average of one-third of the world’s population with the highest prevalence among East Asians [1-4]. The cost involved in correcting myopia is about one-fourth of the entire expenditure in ophthalmology and optometry [2,5]. Its extreme form, high myopia, is the fourth most common cause of irreversible blindness [6,7]. Evidence has demonstrated that genetic factors play an important role in the development of high myopia [1,8-11]. Molecular genetic investigations of high myopia have become a hot topic in recent years. However, the genes responsible for most high myopia are, as yet, unknown.

Expression of *ZENK* (the chicken and mouse ortholog of mammalian *EGR1*, OMIM 128990) has been shown to be involved in ocular growth and refraction. Experiments in animal models have shown that upregulation of *ZENK* in retinal glucagon amacrine cells is assumed to create a STOP signal to inhibit axial eye growth whereas down-regulation of *ZENK* is associated with axial eye growth [12-16]. Therefore, loss/reduction-of-function mutation of *ZENK* might theoretically be associated with myopia. Indeed, *EGR1* knockout mice had longer eyes and a relative myopic shift in refraction [17]. Therefore, it would be interesting to know if there are mutations in *EGR1* of human individuals with myopia especially high myopia where excessive axial elongation of the eye is a common prominent feature.

Here, we analyzed *EGR1* in 96 Chinese patients with high myopia. No mutation was identified in *EGR1*. The results indicate that *EGR1* is unlikely to be responsible for high myopia in these patients.

## Methods

### Subjects

The procedure for collecting subjects and obtaining informed consent was the same as previously described [18]. This study followed the tenets of the Declaration of Helsinki and was approved by the Institutional Review Board. Ophthalmological examinations were performed by ophthalmologists (Q.Z. and X.G.). A total of 96 Chinese subjects with high myopia were recruited who met the following criteria, 1) bilateral refraction of –6.00 D or lower (spherical equivalent) and 2) did not have any other known ocular or systemic disease. The refractive error was measured with cycloplegic autorefraction after mydriasis (Mydrin®-P, a compound tropicamide; Santen Pharmaceutical Co. Ltd., Osaka, Japan) for all eyes. Genomic DNA was prepared from venous blood.

### Mutation detection

Five pairs of primers (Table 1) were used to amplify the two coding exons and the adjacent intronic sequence of the *EGR1* gene (NCBI human genome build 36.3, NC_000005.8 for genomic DNA, NM_001964.2 for mRNA, and NP_001955.1 for protein). DNA sequences of the amplicons were identified with ABI BigDye Terminator cycle sequencing kit version 3.1 (Applied Biosystems, Foster City, CA) on an ABI 3100 Genetic Analyzer (Applied Biosystems). Sequencing results from patients as well as from *EGR1* consensus sequences from the NCBI Human Genome Database (NC_000005.8) were imported into the SeqManII program of the Lasergene package (DNAStar Inc., Madison, WI) and then aligned to identify variations. Each variation was confirmed by bidirectional sequencing. Mutation description followed the recommendation of the Human Genomic Variation Society (HGVS) [19].

**Table 1:** Primers used for polymerase chain reaction amplification and sequencing of *EGR1*.

**Exon**	**Primer sequence (5′-3′)**	**Size of PCR product (bp)**	**Annealing temp (°C)**	**Note**
1	F-GTCGCCGCCTGCACGCTTCT R-AAACCCGGCTCTCATTCTAA	550	64	GC Buffer
2a	F-CCTCGGGAGTCAATGGTAG R-GTGGGTGCCGCTGAGTAAATG	430	63	GC Buffer
2b	F-ACCGGCCTCCTCGTCCTCA R-ACTCCACTGGGCAAGCGTAAG	530	63	
2c	F-CTTCGCTAACCCCTCTGTCTA R-GGTGGCCGGGGATGGATAAG	539	63	
2d	F-TGAACGCAAGAGGCATACCAA R-GGCCATCTCCTCCTCCTGTCC	481	64	GC Buffer

GC buffer was provided by Takara Biotechnology (Dalian) Co. Ltd (Liaoning, China) and is designed for amplification of templates having complex secondary structure or high GC content. In the “Primer sequence” column, “F” indicates the forward sequence and “R” indicates the reverse sequence.

## Results

### Clinical data

A total of 96 unrelated subjects (54 males and 42 females; mean age 17.07±17.01 years) participated in this study. Refraction measures were −12.11±-4.55 D for the right eye and −12.31±-4.81 D for the left eye.

### Mutation analysis

Upon complete sequencing analysis of the coding regions and the adjacent intronic regions, no mutation was identified in the 192 chromosomes of *EGR1* in the 96 subjects with high myopia. One silent variation, c.1026G>A (p.V342V), was detected in one patients with high myopia. This variation would not affect the encoded amino acid.

### Single nucleotide polymorphism analysis

There are three single nucleotide polymorphisms (SNPs) in *EGR1* including rs13181973, rs11953917 (also named rs60458721), and rs1042088. For the 96 subjects with high myopia in this study, only one allele was detected at each of the three SNPs, the C allele for rs13181973, the G allele for rs11953917, and the C allele for rs1042088. This is compatible with the HapMap information for East Asians.

## Discussion

*EGR1* is an early growth response gene that encodes a transcription factor, Egr-1 protein, or ZENK. *ZENK* expression can be upregulated or down-regulated by experimental interference that affects axial eye growth toward hyperopia or myopia in chicken and monkey [12-16]. A knockout of both *EGR1* alleles in C57/BL6 mice leads to longer eyes and a relative myopic shift [17]. However, in human beings, sequence analysis of *EGR1* did not detect any mutation in the 96 patients with high myopia. The results suggest that *EGR1* is unlikely to be the responsible gene for high myopia in these patients.

Apart from *EGR1*, myopia-like changes has been observed in lumican-fibromodulin double null mice [20]. Similarly, no mutation in the *lumican* and *fibromodulin* genes has been identified in families with high myopia [21], although a report claimed an association of myopia with SNPs of *lumican* [22]. The situation in myopia knockout models is different from other disease models such as Leber congenital amaurosis and congenital stationary night blindness where information obtained from animal models is compatible to that from human diseases. In addition, human myopia may be influenced by visual behavior and the visual environment, which may differ greatly between animals and human beings. This may raise the question to what extent does an animal myopia model represent human myopia.

Identification of genes responsible for non-syndromic high myopia is very important but will be very difficult, although several loci for high myopia have been mapped [6,23-32]. Variations in several genes were reported to associate with non-syndromic high myopia [22,33-37]. However, none of these reports have been confirmed by replication study [21,38-42], which is definitely the first priority in association studies [43-47]. Two genes, TGFB-induced factor homeobox 1 (*TGIF)* and *lumican*, have been excluded as candidate genes for high myopia, but they have still been treated as potential candidate genes in several subsequent studies. False-positive results in association studies have been frequently mistreated as useful clues for subsequent studies in recent years, without any replication study. Many researchers may have not realized that most positive associations published (as high as 95%) are false positives [43,45-49]. The most striking problem is the criteria (p<5x10^−2^ or 1x10^−2^) that are inappropriately used to claim an association. The false positive is still rather high even using a more stringent criteria of about 10^−5^ suggested by experts in the field [43,44,49-51]. Genetic association study for complex traits is completely different from mapping a gene for a Mendelian trait where, for the latter, a LOD score of 3 or more (2 or more for X-linked trait) is an indication of statistically significant linkage with a 5% chance of error [52] (this has been well approved by identification of genes responsible for human Mendelian traits in the last two decades). In addition, proper classification of high myopia into two groups, Mendelian trait (familial, congenital, or early onset, moderate to high grade before school age, and myopic fundus change) or complex trait (acquired, late-onset during primary school, slow progress, and minimal or no fundus change), might be helpful in the identification of genes contributing to high myopia [53,54]. It would be very unusual to conduct an association study for Mendelian traits with a prominent heterogeneity such as retinitis pigmentosa, so why is it acceptable to do the same thing for Mendelian high myopia?

## References

[1] Feldkamper M, Schaeffel F (2003). Interactions of genes and environment in myopia.. Dev Ophthalmol.

[2] Choo V (2003). A look at slowing progression of myopia.. Lancet.

[3] McCarty CA, Taylor HR (2000). Myopia and vision 2020.. Am J Ophthalmol.

[4] Fan DS, Lam DS, Lam RF, Lau JT, Chong KS, Cheung EY, Lai RY, Chew SJ (2004). Prevalence, incidence, and progression of myopia of school children in Hong Kong.. Invest Ophthalmol Vis Sci.

[5] Ellwein LB: Updating the Hu 1981 Estimates of the Economic Costs of Visual Disorders and Disabilities. http://www.nei.nih.gov/eyedata/hu estimates.asp#note 2004.

[6] Paluru P, Ronan SM, Heon E, Devoto M, Wildenberg SC, Scavello G, Holleschau A, Makitie O, Cole WG, King RA, Young TL (2003). New locus for autosomal dominant high myopia maps to the long arm of chromosome 17.. Invest Ophthalmol Vis Sci.

[7] Pararajasegaram R (1999). VISION 2020-the right to sight: from strategies to action.. Am J Ophthalmol.

[8] Tang WC, Yap MK, Yip SP (2008). A review of current approaches to identifying human genes involved in myopia.. Clin Exp Optom.

[9] Young TL, Metlapally R, Shay AE (2007). Complex trait genetics of refractive error.. Arch Ophthalmol.

[10] Schaeffel F, Simon P, Feldkaemper M, Ohngemach S, Williams RW (2003). Molecular biology of myopia.. Clin Exp Optom.

[11] Guggenheim JA, Pong-Wong R, Haley CS, Gazzard G, Saw SM (2007). Correlations in refractive errors between siblings in the Singapore Cohort Study of Risk factors for Myopia.. Br J Ophthalmol.

[12] Fischer AJ, McGuire JJ, Schaeffel F, Stell WK (1999). Light- and focus-dependent expression of the transcription factor ZENK in the chick retina.. Nat Neurosci.

[13] Wildsoet CF, Wong RO (1999). A far-sighted view of myopia.. Nat Med.

[14] Bitzer M, Schaeffel F (2002). Defocus-induced changes in ZENK expression in the chicken retina.. Invest Ophthalmol Vis Sci.

[15] Bitzer M, Kovacs B, Feldkaemper M, Schaeffel F (2006). Effects of muscarinic antagonists on ZENK expression in the chicken retina.. Exp Eye Res.

[16] Zhong X, Ge J, Smith EL, Stell WK (2004). Image defocus modulates activity of bipolar and amacrine cells in macaque retina.. Invest Ophthalmol Vis Sci.

[17] Schippert R, Burkhardt E, Feldkaemper M, Schaeffel F (2007). Relative axial myopia in Egr-1 (ZENK) knockout mice.. Invest Ophthalmol Vis Sci.

[18] Zhang Q, Li S, Xiao X, Jia X, Guo X (2007). The 208delG mutation in FSCN2 does not associate with retinal degeneration in Chinese individuals.. Invest Ophthalmol Vis Sci.

[19] den Dunnen JT, Antonarakis SE (2000). Mutation nomenclature extensions and suggestions to describe complex mutations: a discussion.. Hum Mutat.

[20] Chakravarti S, Paul J, Roberts L, Chervoneva I, Oldberg A, Birk DE (2003). Ocular and scleral alterations in gene-targeted lumican-fibromodulin double-null mice.. Invest Ophthalmol Vis Sci.

[21] Paluru PC, Scavello GS, Ganter WR, Young TL (2004). Exclusion of lumican and fibromodulin as candidate genes in MYP3 linked high grade myopia.. Mol Vis.

[22] Wang IJ, Chiang TH, Shih YF, Hsiao CK, Lu SC, Hou YC, Lin LL (2006). The association of single nucleotide polymorphisms in the 5′-regulatory region of the lumican gene with susceptibility to high myopia in Taiwan.. Mol Vis.

[23] Schwartz M, Haim M, Skarsholm D (1990). X-linked myopia: Bornholm eye disease. Linkage to DNA markers on the distal part of Xq.. Clin Genet.

[24] Paluru PC, Nallasamy S, Devoto M, Rappaport EF, Young TL (2005). Identification of a novel locus on 2q for autosomal dominant high-grade myopia.. Invest Ophthalmol Vis Sci.

[25] Young TL, Deeb SS, Ronan SM, Dewan AT, Alvear AB, Scavello GS, Paluru PC, Brott MS, Hayashi T, Holleschau AM, Benegas N, Schwartz M, Atwood LD, Oetting WS, Rosenberg T, Motulsky AG, King RA (2004). X-linked high myopia associated with cone dysfunction.. Arch Ophthalmol.

[26] Young TL, Ronan SM, Alvear AB, Wildenberg SC, Oetting WS, Atwood LD, Wilkin DJ, King RA (1998). A second locus for familial high myopia maps to chromosome 12q.. Am J Hum Genet.

[27] Young TL, Ronan SM, Drahozal LA, Wildenberg SC, Alvear AB, Oetting WS, Atwood LD, Wilkin DJ, King RA (1998). Evidence that a locus for familial high myopia maps to chromosome 18p.. Am J Hum Genet.

[28] Zhang Q, Guo X, Xiao X, Jia X, Li S, Hejtmancik JF (2006). Novel locus for X linked recessive high myopia maps to Xq23-q25 but outside MYP1.. J Med Genet.

[29] Zhang Q, Guo X, Xiao X, Jia X, Li S, Hejtmancik JF (2005). A new locus for autosomal dominant high myopia maps to 4q22-q27 between D4S1578 and D4S1612.. Mol Vis.

[30] Zhang Q, Li S, Xiao X, Jia X, Guo X (2007). Confirmation of a genetic locus for X-linked recessive high myopia outside MYP1.. J Hum Genet.

[31] Nallasamy S, Paluru PC, Devoto M, Wasserman NF, Zhou J, Young TL (2007). Genetic linkage study of high-grade myopia in a Hutterite population from South Dakota.. Mol Vis.

[32] Naiglin L, Gazagne C, Dallongeville F, Thalamas C, Idder A, Rascol O, Malecaze F, Calvas P (2002). A genome wide scan for familial high myopia suggests a novel locus on chromosome 7q36.. J Med Genet.

[33] Han W, Yap MK, Wang J, Yip SP (2006). Family-based association analysis of hepatocyte growth factor (HGF) gene polymorphisms in high myopia.. Invest Ophthalmol Vis Sci.

[34] Inamori Y, Ota M, Inoko H, Okada E, Nishizaki R, Shiota T, Mok J, Oka A, Ohno S, Mizuki N (2007). The COL1A1 gene and high myopia susceptibility in Japanese.. Hum Genet.

[35] Lin HJ, Wan L, Tsai Y, Tsai YY, Fan SS, Tsai CH, Tsai FJ (2006). The TGFbeta1 gene codon 10 polymorphism contributes to the genetic predisposition to high myopia.. Mol Vis.

[36] Tang WC, Yip SP, Lo KK, Ng PW, Choi PS, Lee SY, Yap MK (2007). Linkage and association of myocilin (MYOC) polymorphisms with high myopia in a Chinese population.. Mol Vis.

[37] Lam DS, Lee WS, Leung YF, Tam PO, Fan DS, Fan BJ, Pang CP (2003). TGFbeta-induced factor: a candidate gene for high myopia.. Invest Ophthalmol Vis Sci.

[38] Hasumi Y, Inoko H, Mano S, Ota M, Okada E, Kulski JK, Nishizaki R, Mok J, Oka A, Kumagai N, Nishida T, Ohno S, Mizuki N (2006). Analysis of single nucleotide polymorphisms at 13 loci within the transforming growth factor-induced factor gene shows no association with high myopia in Japanese subjects.. Immunogenetics.

[39] Liang CL, Hung KS, Tsai YY, Chang W, Wang HS, Juo SH (2007). Systematic assessment of the tagging polymorphisms of the COL1A1 gene for high myopia.. J Hum Genet.

[40] Pertile KK, Schache M, Islam FM, Chen CY, Dirani M, Mitchell P, Baird PN (2008). Assessment of TGIF as a candidate gene for myopia.. Invest Ophthalmol Vis Sci.

[41] Li J, Zhang QJ, Xiao XS, Li JZ, Zhang FS, Li SQ, Li W, Li T, Jia XY, Guo L, Guo XM (2003). The SNPs analysis of encoding sequence of interacting factor gene in Chinese population.. Zhonghua Yi Xue Yi Chuan Xue Za Zhi.

[42] Scavello GS, Paluru PC, Ganter WR, Young TL (2004). Sequence variants in the transforming growth beta-induced factor (TGIF) gene are not associated with high myopia.. Invest Ophthalmol Vis Sci.

[43] Colhoun HM, McKeigue PM, Davey Smith G (2003). Problems of reporting genetic associations with complex outcomes.. Lancet.

[44] Dahlman I, Eaves IA, Kosoy R, Morrison VA, Heward J, Gough SC, Allahabadia A, Franklyn JA, Tuomilehto J, Tuomilehto-Wolf E, Cucca F, Guja C, Ionescu-Tirgoviste C, Stevens H, Carr P, Nutland S, McKinney P, Shield JP, Wang W, Cordell HJ, Walker N, Todd JA, Concannon P (2002). Parameters for reliable results in genetic association studies in common disease.. Nat Genet.

[45] Altmuller J, Palmer LJ, Fischer G, Scherb H, Wjst M (2001). Genomewide scans of complex human diseases: true linkage is hard to find.. Am J Hum Genet.

[46] Moonesinghe R, Khoury MJ, Janssens AC (2007). Most published research findings are false-but a little replication goes a long way.. PLoS Med.

[47] Hirschhorn JN, Lohmueller K, Byrne E, Hirschhorn K (2002). A comprehensive review of genetic association studies.. Genet Med.

[48] Ioannidis JP (2005). Why most published research findings are false.. PLoS Med.

[49] Hirschhorn JN, Daly MJ (2005). Genome-wide association studies for common diseases and complex traits.. Nat Rev Genet.

[50] Newton-Cheh C, Hirschhorn JN (2005). Genetic association studies of complex traits: design and analysis issues.. Mutat Res.

[51] Lander E, Kruglyak L (1995). Genetic dissection of complex traits: guidelines for interpreting and reporting linkage results.. Nat Genet.

[52] Terwilliger JD, Ott J. Handbook of human genetic linkage. Baltimore: John Hopkins University Press; 1994.

[53] Morgan IG (2003). The biological basis of myopic refractive error.. Clin Exp Optom.

[54] Morgan I, Rose K (2005). How genetic is school myopia?. Prog Retin Eye Res.

